# The *Plasmodium* serine-type *SERA* proteases display distinct expression patterns and non-essential *in vivo* roles during life cycle progression of the malaria parasite

**DOI:** 10.1111/j.1462-5822.2009.01419.x

**Published:** 2010-01-20

**Authors:** Elyzana D Putrianti, Anja Schmidt-Christensen, Iris Arnold, Volker T Heussler, Kai Matuschewski, Olivier Silvie

**Affiliations:** 1 Parasitology Unit, Max Planck Institute for Infection Biology10117 Berlin, Germany; 2 Department of Parasitology, Heidelberg University School of Medicine69120 Heidelberg, Germany; 3 Bernhard Nocht Institute for Tropical Medicine, Department of Molecular Parasitology20359 Hamburg, Germany

## Abstract

Parasite proteases play key roles in several fundamental steps of the *Plasmodium* life cycle, including haemoglobin degradation, host cell invasion and parasite egress. *Plasmodium* exit from infected host cells appears to be mediated by a class of papain-like cysteine proteases called ‘serine repeat antigens’ (SERAs). A SERA subfamily, represented by *Plasmodium falciparum* SERA5, contains an atypical active site serine residue instead of a catalytic cysteine. Members of this SERAser subfamily are abundantly expressed in asexual blood stages, rendering them attractive drug and vaccine targets. In this study, we show by antibody localization and *in vivo* fluorescent tagging with the red fluorescent protein mCherry that the two *P. berghei* serine-type family members, *PbSERA1* and *PbSERA2*, display differential expression towards the final stages of merozoite formation. Via targeted gene replacement, we generated single and double gene knockouts of the *P. berghei SERAser* genes. These loss-of-function lines progressed normally through the parasite life cycle, suggesting a specialized, non-vital role for serine-type *SERAs in vivo*. Parasites lacking *PbSERAser* showed increased expression of the cysteine-type *PbSERA3*. Compensatory mechanisms between distinct SERA subfamilies may thus explain the absence of phenotypical defect in *SERAser* disruptants, and challenge the suitability to develop potent antimalarial drugs based on specific inhibitors of *Plasmodium* serine-type SERAs.

## Introduction

Intracellular pathogens have evolved numerous strategies to exit their host cells after completion of replication and growth and depletion of host cell nutrients (Hybiske and Stephens, 2008). Cellular exit is often an active biological process triggered by the pathogen and accompanied by consecutive breaching of the membrane of the parasitophorous vacuole (PV) that harbours the pathogen and the host cell plasma membrane.

*Plasmodium* and other apicomplexan parasites are obligate intracellular pathogens that need to efficiently enter and exit their respective host cells in order to propagate and progress along the life cycle. Studies with broad-spectrum cysteine inhibitors have indicated central roles for proteolytic events during egress of merozoites, the invasive stage of the malarial parasite in the pathogenic red blood cell cycle, out of the PV and the erythrocyte plasma membrane (Salmon *et al.*, 2001; Wickham *et al.*, 2003). *Plasmodium* appears to compartmentalize proteins that function specifically in parasite egress in specialized electron-dense secretory organelles termed ‘exonemes’ (Yeoh *et al.*, 2007). Exonemes contain the subtilisin-like serine protease subtilase 1 (SUB1) that is essential for parasite growth and can proteolytically activate a family of papain-like proteases termed ‘serine-repeat antigens’ (SERAs), which in turn may mediate parasite egress through subsequent processing of cellular substrates. Therefore, exoneme discharge may trigger a proteolytic cascade that ultimately leads to cytolysis and parasite exit (Yeoh *et al.*, 2007). Understanding the cellular roles of SERAs, which constitute major substrates of SUB1, may ultimately lead to the identification of parasite and/or host cell substrates and the underlying molecular mechanisms of proteolysis. Direct support for the proposed roles for SERAs in parasite egress comes from experimental genetics. Loss of *PbSERA5/ECP1* function results in viable and motile sporozoites that are defective in exiting the midgut oocyst in the insect vector (Aly and Matuschewski, 2005). Remarkably, members of the *SERA* multigene family appear to have arisen from multiple gene duplication events. In *P. falciparum* eight out of nine *SERA*s are located in tandem on chromosome 2 (Aoki *et al.*, 2002; Miller *et al.*, 2002). Similarly, in the rodent malaria model parasite *P. berghei* the five *SERAs* are tandemly arranged in a head-to-tail fashion on chromosome 3 (Kooij *et al.*, 2005).

This gene organization is evolutionary conserved and is a hallmark of the *SERA* multigene family (Arisue *et al.*, 2007; McCoubrie *et al.*, 2007).

All *Plasmodium* SERAs contain a central, papain-like protease domain and numerous cysteine residues. Intriguingly, this class of proteins appears to be absent in a number of related apicomplexan parasites, such as *Toxoplasma gondii* or *Cryptosporidium parvum*, suggesting that their respective roles are restricted to malaria parasites. Despite their overall sequence similarity in their central protease domain, *Plasmodium* SERA proteins can be classified into four major groups that form two distinct phylogenetic clusters (Hodder *et al.*, 2003; Kooij *et al.*, 2005; Arisue *et al.*, 2007; McCoubrie *et al.*, 2007). The active site cysteine SERAs (SERAcys) form three separate groups within one cluster, whereas those with an active site serine (SERAser) form a fourth monophyletic group.

The three orthologous SERAcys groups appear to be well conserved across the genus *Plasmodium*. Two groups, represented by *P. falciparum PfSERA6* and *PfSERA7*, respectively, are expressed in asexual parasites, whereas the third and most ancestral group, represented by *PfSERA8*, is not (Aoki *et al.*, 2002; Miller *et al.*, 2002). Targeted gene deletion of the *P. berghei* orthologue of *PfSERA8*, termed ‘egress cysteine protease 1’ (*ECP1*), confirmed a dispensable role in the mammalian host and instead revealed an essential function for sporozoite egress from oocysts in the mosquito vector (Aly and Matuschewski, 2005). By analogy, members of the *PfSERA6* and *PfSERA7* groups may function in parasite egress out of mammalian host cells.

In contrast, the cellular roles of SERAser proteins, which together form the most diverse group, remain largely unsolved. The founding member *Pf*SERA5 localizes to the PV of mature schizonts (Delplace *et al.*, 1987; Miller *et al.*, 2002). Purified recombinant *Pf*SERA5 protein exhibits only limited chymotrypsin-like autoproteolytic activity and cleavage of polypeptide substrates is negligible (Hodder *et al.*, 2003). However, this group stands apart, because (i) expression analysis revealed that *SERAser* genes, particularly *SERA5*, are very abundantly expressed in *P. falciparum* late trophozoites and schizonts, the parasite stages preceeding parasite egress from their host erythrocytes (Aoki *et al.*, 2002; Lasonder *et al.*, 2002; Miller *et al.*, 2002), (ii) antibodies against *Pf*SERA5 inhibit parasite erythrocytic growth *in vitro* through agglutination of merozoites and ruptured schizonts (Pang *et al.*, 1999), (iii) infected individuals in malaria-endemic areas exhibit high antibody titres against SERAser proteins, and most prominently SERA5 (Okech *et al.*, 2001; Aoki *et al.*, 2002; Okech *et al.*, 2006), and (iv) high anti-*Pf*SERA5 antibody titres correlate with protection against severe disease (Okech *et al.*, 2006). In a monkey model immunization with a purified recombinant *Pf*SERA5 fragment induces protection against challenge infection (Inselburg *et al.*, 1991). Therefore, SERAser proteins represent the most promising group of all SERAs for potential therapeutic and vaccine targets.

In this study, we investigated the cellular roles of *SERAser* by experimental genetics in the model rodent malaria parasite *P. berghei*. Unexpectedly, we could exclude an essential role for all *SERAser* during the *P. berghei* life cycle. Our data suggest that this monophyletic *SERA* group evolved in the absence of vital roles for the parasite.

## Results

### *Plasmodium berghei* serine-type SERA proteases

The model rodent malaria parasite *P. berghei* encodes two members of the SERAser subfamily, *Pb*SERA1 and *Pb*SERA2 (Aly and Matuschewski, 2005; Kooij *et al.*, 2005).

**Fig. 1:**
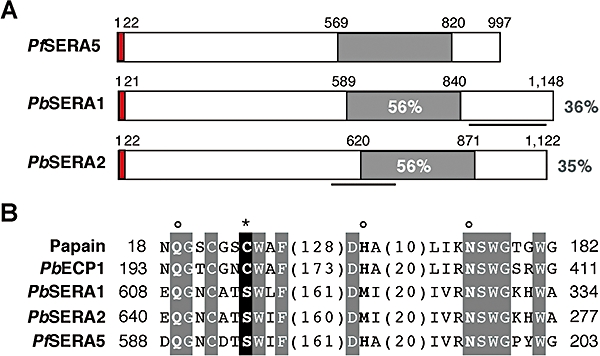
The rodent malaria active-site serine SERA subfamily. A. Primary structure of *Plasmodium* SERA proteins with active site serines (SERAser). The central papain-like cysteine protease domains are boxed in grey. Overall amino acid sequence identities of the *P. berghei* SERA1 (EU917224) and SERA2 (EU917225) sequences are indicated as percentages of identical residues compared with the *P. falciparum* SERA5 (PFB0340c). Fragments used for protein expression, purification and antibody production are indicated by bars. B. Partial conservation of the catalytic residues of the papain family within the central serine protease domain. *P. berghei* SERA1 and SERA2 and *P. falciparum* SERA5 are shown together with papain and a *P. berghei* cysteine family SERA protein, *Pb*ECP1/SERA5. The strictly conserved amino acid residues are boxed in grey, and the putative active-site serine or cysteine is shown in bold and boxed in black. Additional residues of the active site are marked with an ‘o’. Note that the catalytic histidine is replaced by a methionine. In contrast, the carboxy-terminal asparagine and the amino-terminal glutamine, which form the oxyanione hole, are conserved.

Direct sequencing of cDNA from asynchronous blood stages permitted identification of the complete coding sequences (GenBank accession numbers: EU917224 and EU917225 for *Pb*SERA1 and *Pb*SERA2 respectively). Comparison of the *P. berghei* orthologues with the founding member *Pf*SERA5 (PFB0340c) illustrates the overall amino acid sequence similarity (Fig. 1A) of ∼35% to the human malaria protein. A hallmark of this subfamily is the replacement of the catalytically active cysteine residue by a corresponding serine residue (Fig. 1B). The other amino acids of the catalytic centre, i.e. an amino-terminal glutamine and a carboxyterminal asparagine, are well conserved, apart from a histidine residue, which is changed to a methionine residue in the homologues of rodent malaria parasites (Hodder *et al.*, 2003; Arisue *et al.*, 2007; McCoubrie *et al.*, 2007).

**Fig. 2:**
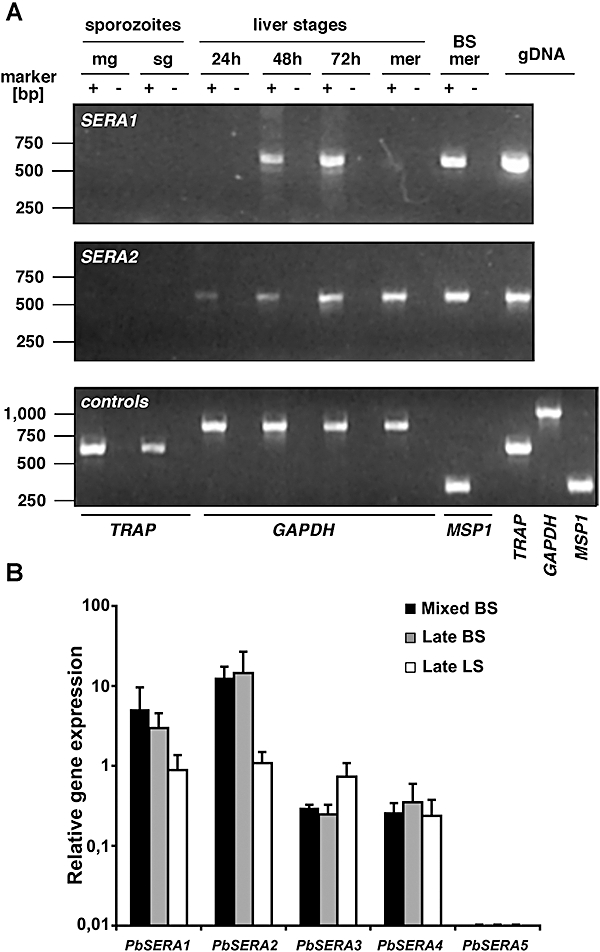
Expression profiling of *P. berghei SERAser*. A. RT-PCR analysis of *SERA1* and *SERA2* expression in mosquito midgut (mg) and salivary gland (sg) sporozoites, mid (24 h) and late (48 h, 72 h) liver stages, liver stage merosomes (mer) and blood stage merozoites (BS mer). The merozoite and sporozoite-specific transcripts, *MSP1* and *TRAP*, and the constitutive *GAPDH* transcript were added as controls. cDNAs were synthesized from mRNA in the presence (+) or absence (−) of reverse transcriptase. Genomic DNA (gDNA) was added as an amplification control. B. Quantitative RT-PCR analysis of *P. berghei SERA* gene expression in WT mixed blood stages (BS), purified schizonts (late BS) or infected HuH7 cell cultures 65 h post-infection (late LS). Relative gene expression was normalized to *MSP1* expression level, and is shown as the mean of two independent experiments (±SD).

In order to initiate a genetic characterization of the *SERAser* subfamily, we profiled their expression by RT-PCR analysis (Fig. 2A). In good agreement with *P. falciparum* expression data (Aoki *et al.*, 2002; Miller *et al.*, 2002), both transcripts are readily detectable in blood stage merozoites. Both transcripts are absent during sporozoite maturation and expression commences again during liver stage development. Both *PbSERA1* and *PbSERA2* appear to be abundantly expressed late in liver stage development (Fig. 2A), as observed previously (Schmidt-Christensen *et al.*, 2008). Together, these data suggest that the two *SERAser* genes are expressed during formation of liver stage and blood stage merozoites.

We further quantified the relative transcript abundance for the five *P. berghei SERA* genes using real-time RT-PCR (Fig. 2B). The two *SERAser* genes, *PbSERA1* and *PbSERA2*, were the most abundantly expressed in blood stages. In late liver stages, *PbSERA1*, *PbSERA2* and *PbSERA3* were expressed at a similar level, whereas *PbSERA4* transcripts were less abundant. As expected (Aly and Matuschewski, 2005), *PbSERA5* was not expressed in liver or blood stages (Fig. 2B). These data confirm that the *SERAser* genes (and particularly *PbSERA2*) are the *SERAs* most prominently expressed in blood stages, consistent with *P. falciparum* expression data (McCoubrie *et al.*, 2007).

### Cellular localization of *Pb*SERA1 and *Pb*SERA2

**Fig. 3:**
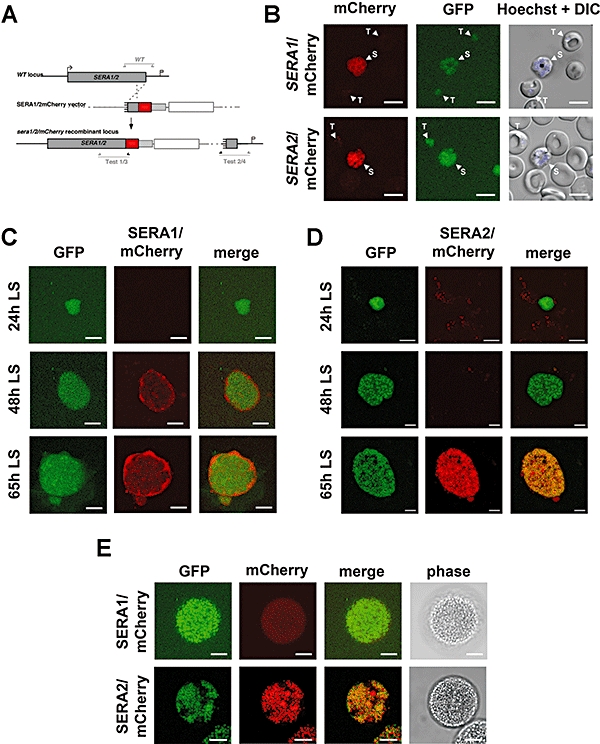
Fluorescent tagging of *P. berghei SERA1* and *SERA2.* A. Insertion strategy to generate the SERA1/mCherry and SERA2/mCherry parasites. The *PbSERA1* and *PbSERA2* genomic loci were targeted with integration plasmids containing the 3′*SERA1* and *SERA2* terminal fragments (dark grey box) that is fused in frame to the mCherry coding sequence (red box), the 3′ UTR of *PbDHFR/TS* (light grey box) and the *TgDHFR/TS* selectable marker (white box). Upon a single cross-over event, the region of homology is duplicated, resulting in a functional, endogenous *PbSERA1* or *PbSERA2* copy tagged with mCherry, followed by a truncated and non-expressed copy. B. Expression of the mCherry fusion proteins (red) was analysed by confocal fluorescence microscopy of SERA1/mCherry and SERA2/mCherry *P. berghei* blood stage parasites constitutively expressing GFP (green). Parasite stages are indicated by arrow heads. T, ring/trophozoite; S, schizont. Nuclei were stained with Hoechst 33342. Bars, 5 µm. C and D. Liver stage expression of the mCherry fusion proteins (red) was analysed by confocal fluorescence microscopy of SERA1/mCherry (C) and SERA2/mCherry (D) *P. berghei* parasites constitutively expressing GFP (green), at 24, 48 and 65 h after infection of HuH7 cells with sporozoites. Bars, 10 µm. E. Detached infected cells (merosomes) were recovered from the supernatant of infected HuH7 cultures 65 h post infection, and analysed by confocal fluorescence microscopy. Size bars, 10 µm.

We next investigated the localization and the expression timing of *Pb*SERA1 and *Pb*SERA2. For this purpose we generated parasite lines expressing the endogenous SERA1 and SERA2 proteins fused to the mCherry red fluorescent protein (Shaner *et al.*, 2004). This was achieved by transfection of targeting vectors that contain an amino-terminally truncated *PbSERA1* or *PbSERA2* copy and in-frame fusion of the mCherry coding region, followed by the *DHFR/TS* 3′ untranslated region (Fig. 3A). Upon a single cross-over event, integration of these constructs is predicted to result in an allelic duplication, resulting in a mCherry-tagged full-length copy and a non-transcribed 5′ truncated version of the *PbSERA1* or *PbSERA2* gene.

Transfection was performed in *P. berghei* ANKA parasites expressing GFP (Janse *et al.*, 2006), leading to green fluorescent parasites that express a red fluorescent SERA1 or SERA2 protein. Genotyping by PCR using specific primer combinations confirmed the desired integration events (Fig. S1).

We first performed live cell imaging of blood stages of the transgenic *PbSERA1/mCherry* and *PbSERA2/mCherry* parasites. Similarly to SERA5 in *P. falciparum* (Delplace *et al.*, 1987), both *Pb*SERA1/mCherry and *Pb*SERA2/mCherry were detected in late schizonts, but not in early blood stages (Fig. 3B). *PbSERA1/mCherry* and *PbSERA2/mCherry* parasites developed normal asexual and sexual blood stages, and could be transmitted to *Anopheles* mosquitoes, resulting in the formation of sporozoites (data not shown). In good agreement with our transcription analysis, *Pb*SERA1/mCherry and *Pb*SERA2/mCherry were barely detectable during the mosquito stages (data not shown).

We next investigated expression of mCherry-tagged SERAs during liver stage development *in vitro*. As observed with blood stages, the fusion proteins were not detected in early liver stages, but were abundantly expressed in late liver stages (Fig. 3C and D). Remarkably, *Pb*SERA1/mCherry and *Pb*SERA2/mCherry showed clearly distinct expression patterns in liver stages. *Pb*SERA1/mCherry was detected in mid and late liver stages, and localized predominantly to the PV, which constitutes the parasite/host interface (Fig. 3C). In contrast, *Pb*SERA2/mCherry became detectable only at the end of liver stage development, with an intracellular distribution in the parasite (Fig. 3D). Interestingly, *Pb*SERA1/mCherry was not detected in merosomes, in contrast to *Pb*SERA2/mCherry, which gave a strong signal associated with individual merozoites inside merosomes (Fig. 3E). Collectively, these data indicate that both *Pb*SERA1 and *Pb*SERA2 are expressed in late blood and liver stages. Importantly, the two proteins apparently distribute to distinct compartments, and only *Pb*SERA2 remains associated with merozoites after PV membrane (PVM) rupture.

**Fig. 4:**
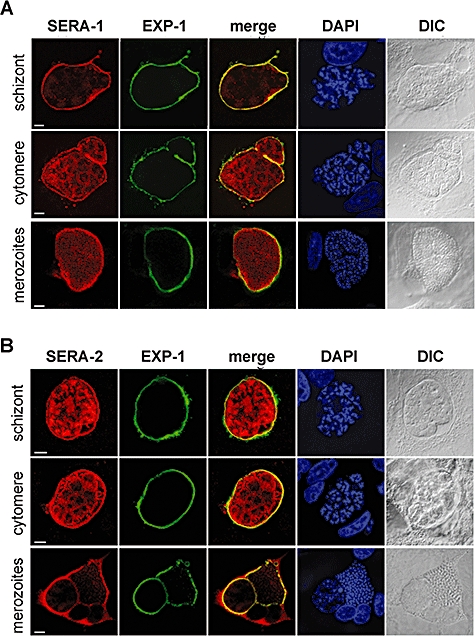
Immunofluorescence analysis of *Pb*SERA1 and *Pb*SERA2 in late liver stages. *P. berghei* infected HepG2 cells were fixed at different time points (45–58 h) post infection and analysed on single cell level using IFA. Developmental stages are indicated on the left. Localization of the C-terminal region of *Pb*SERA1 (A) and the central domain of *Pb*SERA2 (B) were determined using specific rat antibodies (red). As a marker for the PVM, we used chicken anti-EXP1 antibodies (green), and DNA was labelled with DAPI (blue). Scale bar: 5 µm. Note the distribution of SERA1C to the PV compartment in all stages, whereas SERA2M localizes both to the PV and internal parasite structures in schizonts and cytomeres, but only to the PV compartment after merozoite differentiation.

To get further insights into the distribution of *Pb*SERAser proteins, we performed immunofluorescence analysis of late liver stages, using antibodies generated against the C-terminus of *Pb*SERA1 (anti-SERA1C) or the central domain of *Pb*SERA2 (anti-SERA2M) respectively (Fig. 1A). In late liver stages, staining with anti-SERA1C antibodies was mostly restricted to the PVM, as shown by colocalization with the PVM marker exported protein 1 (EXP1) (Fig. 4A, upper panels). This pattern was also observed at more advanced stages of development, in cytomeres and fully differentiated merozoite-containing parasites (Fig. 4A, middle and lower panels). This distribution is reminiscent of the fluorescence pattern observed in parasites harbouring a mCherry tag at the C-terminus of *Pb*SERA1 (Fig. 3C). In contrast, anti-SERA2M antibodies showed a more complex distribution in late liver schizonts and cytomeres, staining both the PVM and more internal structures (Fig. 4B, upper and middle panels).

Interestingly, in terminal liver stages, just prior to the release of merozoites, SERA2M was detected at the periphery of the parasites, in the PV and/or PVM compartment, as well as the host cell cytoplasm (Fig. 4B, lower panels). This distribution differs from that of the C-terminal fragment of SERA2, based on the fluorescence pattern in *Pb*SERA2/mCherry parasites (Fig. 3D). Together, these results strongly suggest that SERA2 is processed towards the end of liver stage development, with the central putative papain-like domain being released in the vacuolar space, while the C-terminal domain (visualized by the mCherry tag) remains associated with the merozoites. In good agreement with this hypothesis, Western blot analysis of purified blood stage schizonts demonstrated that SERA2 processed forms were enriched in saponin extracts, as compared with Triton X-100 (Fig. S2), consistent with localization to the PV compartment of cleaved products that contain the central domain recognized by the anti-*Pb*SERA2M antibodies.

### Generation of *Pb*SERA1 and *Pb*SERA2 knockout parasites

**Fig. 5:**
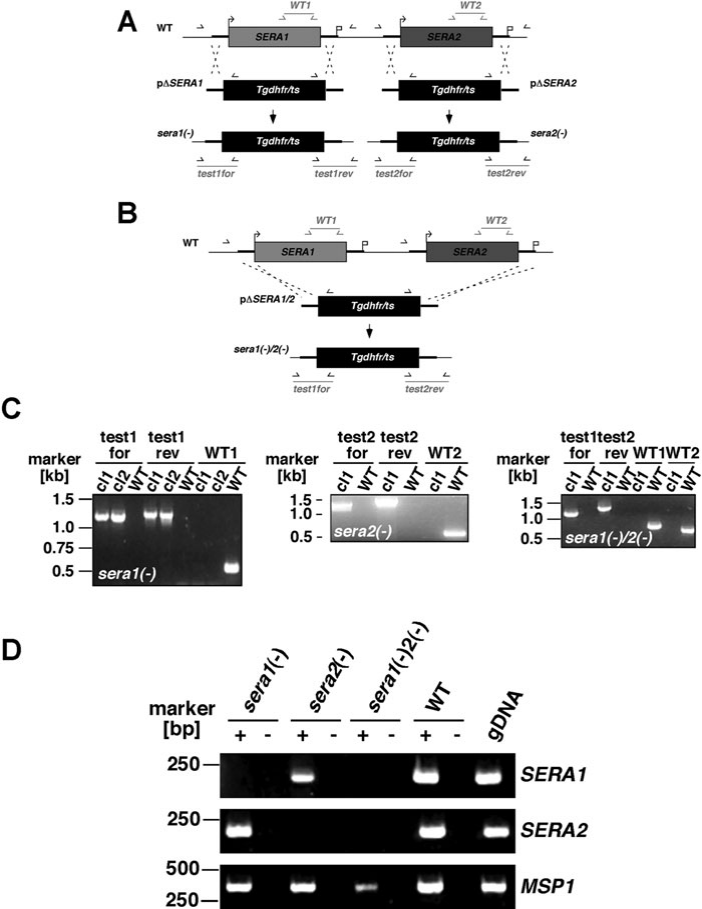
Targeted gene disruption of the *P. berghei SERAser* genes. A and B. Replacement strategy to generate the *sera1*(−) and *sera2*(−) parasites (A), and the *sera1*(−)/*2*(−) parasites (B). The wild-type (WT) *SERAser* genomic loci are targeted with KpnI*/*SacII-linearized replacement plasmids (pREP) containing 5′ and 3′ untranslated regions adjacent to the *SERAser* open reading frames and the *dhfr/ts*-positive selectable marker. Upon a double cross-over event the open reading frame is replaced by the selectable marker. Replacement-specific test and WT primer combinations are indicated by arrows and expected fragments as lines. C. Replacement-specific PCR analysis. The successful replacement event is verified by a primer combination (test) that can only amplify a signal from the *REP* locus. Absence of the WT signal from *sera1*(−), *sera2*(−) and *sera1*(−)/2(−) parasites confirms the purity of the clonal populations. D. RT-PCR analysis of *SERA1*, *SERA2* and *MSP1* transcripts in WT, *sera1*(−), *sera2*(−) and *sera1*(−)/2(−) blood stage parasites. RT-PCR was performed in the presence (+) or absence (−) of reverse transcriptase (RT). Parasite genomic DNA (gDNA) was included as an amplification control.

**Fig. 6:**
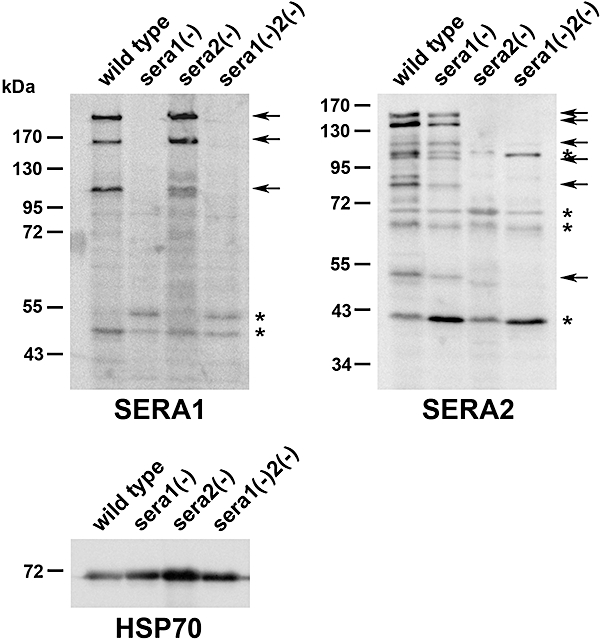
Western blot analysis of PbSERA1 and PbSERA2 protein expression in *P. berghei* blood stages. Triton X-100 extracts of enriched schizont preparations from WT, *sera1*(−), *sera2*(−) and *sera1*(−)*2*(−) parasites were analysed by Western blot using antibodies specific for *Pb*SERA1 and *Pb*SERA2. Anti-HSP70 antibodies were used as a loading control. Note the absence of specific bands (arrows) in the knockout parasite lines, confirming complete gene disruption. Non-specific bands are indicated with an asterisk.

We next wanted to study the cellular functions of *Pb*SERA1 and *Pb*SERA2 in the *Plasmodium* life cycle. Based on the refractoriness to gene knockout of *PfSERA5* (McCoubrie *et al.*, 2007), we expected that at least one member of the *P. berghei SERAser* family plays a vital role during asexual growth and therefore cannot be targeted by classical reverse genetics. We generated replacement vectors that were designed to disrupt the corresponding open reading frames by double homologous recombination after transfection into *P. berghei* parasites (Fig. 5A). Unexpectedly, we could select recombinant parasite populations with the antifolate pyrimethamine. Subsequent cloning of single parasites resulted in multiple clonal *sera1*(−) and *sera2*(−) parasite lines. Genotyping by PCR using specific primer combinations confirmed the expected recombination events in *sera1*(−) and *sera2*(−) parasite lines (Fig. 5C), and RT-PCR demonstrated the complete absence of *SERA1* transcripts and *SERA2* transcripts in *sera1*(−) and *sera2*(−) blood stage parasites respectively (Fig. 5D). Furthermore, Western blot analysis of purified blood schizonts demonstrated the absence of *Pb*SERA1 and *Pb*SERA2 proteins in *sera1*(−) and *sera2*(−) blood stage parasites respectively (Fig. 6). Collectively, these results confirm the successful disruption of the corresponding gene.

### *Pb*SERA1 and *Pb*SERA2 are dispensable during the parasite life cycle

We then analysed the phenotypes of *sera1*(−) and *sera2*(−) parasites. The successful disruption of *SERA1* and *SERA2* genes in *P. berghei* blood stages indicated that both are dispensable during blood stage multiplication of the parasite. Both *sera1*(−) and *sera2*(−) parasites produced gametocytes and exflagellation of male gametocytes was similar to WT parasites (data not shown). Transmission to *Anopheles stephensi* mosquitoes and oocyst development were also normal when compared with WT parasites (Table 1). Both *sera1*(−) and *sera2*(−) oocysts produced sporozoites, which invaded mosquito salivary glands as efficiently as WT parasites (Table 1). These findings demonstrate that *SERA1* and *SERA2* are dispensable for the *P. berghei* life cycle in the mosquito vector, in good agreement with the absence of gene expression in the mosquito stages (Fig. 2A). Sporozoites from *sera1*(−) and *sera2*(−) parasites displayed normal gliding motility (Table 1), and were infective to rats (Table 2). Importantly, after intravenous injection of sporozoites or administration through mosquito bites, the natural transmission route, we observed no delay in patency as compared with WT parasites (Table 2). This clearly shows that hepatic merozoites are formed and released normally in *sera1*(−) and *sera2*(−) *P. berghei* parasites. This was confirmed by *in vitro* experiments, which demonstrated that *sera1*(−) and *sera2*(−) parasites form exoerythrocytic forms (EEFs) in cultures, in numbers comparable with WT parasites (Table 1). Together, these findings demonstrate that individual *SERAser* are dispensable during the *P. berghei* life cycle.

**Table 2:** Infectivity of *SERAser(*−*)* mutants to rats.

Parasite population	Sporozoite dose^a^	No. infected animals/No. injected animals	Prepatency (days)^b^
WT	10 000	8/8	3.3
	Mosquito bite	2/2	3.0
*sera1(*−*)*	10 000	3/3	3.3
*sera2(*−*)*	10 000	2/2	3.5
	25 000	2/2	3.5
	50 000	1/1	3.0
	Mosquito bite	1/1	3.0
*sera1(*−*)/2(*−*)*	10 000	5/5	3.4

a Sporozoites were either injected intravenously at the doses indicated or delivered by natural mosquito bite via exposure of anesthetized SD rats to 5 infected *Anopheles* mosquitoes.

b Prepatency is the time until the first detection of an erythrocytic stage parasite in Giemsa-stained thin blood smears after sporozoite infection.

**Table 1:** Phenotypic analysis of *SERAser(*−*)* mutants.

			Mean no. of sporozoites/ infected mosquito				
Experiment	Parasite population	Infectivity	Midgut	Salivary glands	Gliding motility^a^	EEF 24 h	EEF 48 h
I	WT	95%	47 000	23 500	++	222 (±16)	170 (±24)
	*sera1(*−*)*	90%	33 300	30 550	++	200 (±04)	161 (±17)
II	WT	85%	17 500	11 200	++	235 (±19)	131 (±12)
	*sera2(*−*)*	65%	38 100	8 050	++	113 (±12)	96 (±15)
	*sera1(*−*)/2(*−*)*	90%	34 550	16 600	++	242 (±05)	112 (±22)
III	WT	85%	31 500	14 000	++	237 (±11)	102 (±08)
	*sera1(*−*)/2(*−*)*	75%	36 750	11 400	++	229 (±28)	102 (±19)

a Gliding motility was visualized by CSP labelling of salivary gland sporozoites on glass slides, and motile sporozoites were counted using a fluorescence microscope.

++, continuous, multiple trails in more than 30% of sporozoites.

### *P. berghei* lacking both *SERAser* progress normally through the parasite life cycle

We hypothesized that compensatory mechanisms within the SERAser subfamily may explain the absence of phenotypical defect in single gene disruptants. Therefore, we generated parasite lines with a double *PbSERA1* and *PbSERA2* gene deletion. To this end, we used a replacement vector containing the 5′ region of *PbSERA1*, the selection cassette and the 3′ region of *PbSERA2* (Fig. 5B). Remarkably, after transfection of *P. berghei* parasites with this targeting construct, we could select and isolate *sera1*(−)/*2*(−) parasite populations. Genotyping by PCR using specific primer combinations confirmed the expected recombination events (Fig. 5C). Further more, RT-PCR and Western blot analysis demonstrated the complete absence of *SERA1* and *SERA2* transcripts (Fig. 5D) and proteins (Fig. 6), respectively, in *sera1*(−)/*2*(−) blood stage parasites, confirming the simultaneous disruption of both *SERA1* and *SERA2* genes. As expected, immunofluorescence analysis confirmed the absence of staining of *sera1*(−)/*2*(−) late liver stages with anti-SERA1 and anti-SERA2 antibodies, which also demonstrates the specificity of the staining pattern observed in WT parasites with these antibodies (Fig. S3 and Fig. 4).

**Fig. 7:**
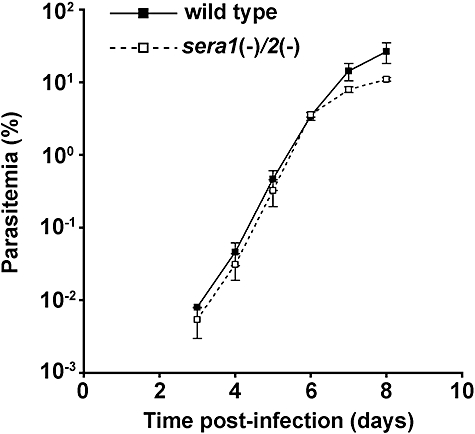
Blood stage growth of *sera1*(−)/*2*(−) double knockout *P. berghei* parasites. Naïve NMRI mice (*n* = 5) were injected intravenously with 1000 wild-type or 1000 *sera1*(−)/*2*(−) *P. berghei* infected erythrocytes. Infection was then monitored by daily examination of Giemsa-stained blood smears to determine the parasitemia.

As observed with the single gene mutants, *sera1*(−)/*2*(−) displayed no obvious defect during asexual blood stage growth (Fig. 7) and sexual stage differentiation (data not shown). The double disruptants could be transmitted to mosquitoes, where parasite development was not affected (Table 1). *sera1*(−)/*2*(−) parasites produced normal numbers of sporozoites (Table 1), which were motile and as infective to rodents as WT parasites (Table 2). As observed with single gene disruptants, *sera1*(−)/*2*(−) parasites formed EEFs *in vitro*, in similar numbers as WT (Table 1). Furthermore, the number of detached infected cells released in culture supernatants was similar between WT (473 ± 200 merosomes per well) and *sera1*(−)/*2*(−) parasites (433 ± 250 merosomes per well), and mice injected with 500 WT merosomes (n=3) or *sera1*(−)/*2*(−) merosomes (n=3) all developed a patent blood stage infection at day 2 post injection. Together, these data establish that in the absence of *SERAser*, *P. berghei* parasites progress normally through their life cycle. In particular, parasites lacking *SERA1* and *SERA2* display no detectable defect in egress from infected host cells.

### *P. berghei* lacking *SERAser* display increased expression of the cysteine-type SERA3

**Fig. 8:**
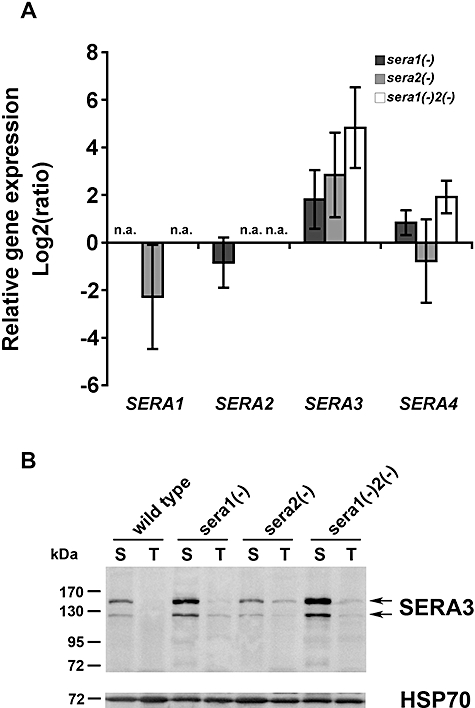
Upregulation of *PbSERA3* expression in *SERAser* knockout lines. A. *SERA* gene expression was analysed by quantitative RT-PCR in purified schizonts from WT, *sera1*(−), *sera2*(−) and *sera1*(−)*/2*(−) parasites. Relative gene expression was normalized to *MSP1* expression level, and is shown as the Log2 of the ratio knockout/WT (mean of two experiments ± SD). n.a., no amplification. B. Saponin (S) or Triton X-100 (T) extracts of purified schizonts from WT, *sera1*(−), *sera2*(−) and *sera1*(−)*/2*(−) parasites were analysed by Western blot using antibodies specific for *Pb*SERA3. As a loading control an anti-HSP70 antibody was used.

Finally, we analysed the impact of *SERAser* gene disruption on the expression of the cysteine-type *SERAs PbSERA3* and *PbSERA4*. Quantitative PCR on cDNA prepared from purified blood schizonts showed a modest reduction of SERA1 transcript levels in *sera2*(−) parasites, whereas SERA2 transcript levels were not significantly modified in *sera1*(−) parasites. In sharp contrast, in both *sera1*(−) and *sera2*(−) parasites there was an upregulation of *PbSERA3*, but not *SERA4*, expression (Fig. 8A). The increase in *PbSERA3* transcript levels was even more pronounced in parasites lacking both *SERAser* (Fig. 8A). To confirm a compensatory upregulation of *Pb*SERA3 in the *SERAser* loss-of-function parasite lines we analysed the protein levels in merozoites (Fig. 8B). *Pb*SERA3 was detected mainly in saponin extracts, confirming its localization to the PV compartment in blood stages, as reported previously (Schmidt-Christensen *et al.*, 2008). In good agreement with the transcript profiling we detected an increase in *Pb*SERA3 protein by 2- and 2.5-fold in *sera1*(−) and *sera1*(−)/2(−) parasites respectively. These results indicate a potential functional link between distinct SERA subfamilies, and provide a possible explanation for the absence of phenotypical defect in *PbSERAser*-deficient parasites.

## Discussion

The most important finding from our study is the non-essential role of the *SERAser* subfamily *in vivo* in the model rodent malaria parasite *P. berghei*. We successfully deleted the single genes of *SERA1*, *SERA2* and generated a *SERA1/2* double knockout. Both single mutants and the double mutant showed no apparent defect at any phase of the *Plasmodium* life cycle under standard conditions. Most importantly, the successful generation of the double mutant demonstrates that redundancy is not an essential feature of the *SERAser* family *in vivo*. This finding was unexpected, because *SERAser* members are abundantly expressed in asexual blood stages (Aoki *et al.*, 2002; Miller *et al.*, 2002) and at least one member of the *P. falciparum SERAser* family, *SERA5*, appears to be refractory to targeted gene deletion under *in vitro* culture conditions (Miller *et al.*, 2002; McCoubrie *et al.*, 2007). Our findings suggest that the monophyletic group of SERAser enzymes serves a specialized and non-essential role across the genus *Plasmodium*. Our experimental genetics evidence is supported by the *SERA* phylogeny (Arisue *et al.*, 2007; McCoubrie *et al.*, 2007). Based on available genome sequence data, the avian parasite *P. gallinaceum* encodes only for SERAcys members and appears to have diverged from a common *Plasmodium* ancestor prior to a gene duplication event that formed the two separate phylogenetic clusters. In good agreement, we now show that the rodent malaria parasite does not rely on *SERAser* genes for parasite life cycle progression. We propose that members of this monophyletic group evolved gradually and independent of strong selection pressure for parasite growth. However, although SERAser do not play any vital function in rodent malaria parasites, we cannot exclude a role of immunological pressure in evolution of this family, including its expansion in the human parasites.

Notably, one important feature distinguishes the *P. falciparum* SERAser proteins from their other relatives (Hodder *et al.*, 2003). In their central domain they retain a histidine residue that is thought to be part of the catalytic triad. This residue is generally substituted by leucine in *P. vivax* and *P. knowlesi* SERAser proteins, except for *P. vivax* SERA2/*P. knowlesi* SERA5, and by methionine in the rodent *Plasmodium* species. Hence, we currently cannot formally exclude distinct roles of the histidine-containing *P. falciparum* proteins, although their *in vitro* proteolytic activity is weak (Hodder *et al.*, 2003). This possibility can now be addressed experimentally by generating a *trans*-species complementation of the *PfSERA5* deletion that consistently appears to be required for *P. falciparum* growth under *in vitro* culture conditions (Miller *et al.*, 2002; McCoubrie *et al.*, 2007). If complementation with *PbSERA1* or *PbSERA2* rescues the growth defect, an essential role for the histidine residue, and perhaps the *PfSERA5* gene as a whole, can be excluded *in vivo*, and vice versa. Because of the absence of an apparent phenotype in the *PbSERA1*(−)*/2*(−) double mutant, the reverse experiment, i.e. complementation with *PfSERA5*, would not be informative. Although indirect, such an approach will eventually substantiate an important *in vivo* role for *PfSERA5*.

Our expression and localization studies with specific antibodies and fluorescently tagged parasite lines show that *Pb*SERA1 and *Pb*SERA2 have distinct expression patterns, both in terms of expression timing and subcellular localization. After proteolytic processing, N-terminal and C-terminal parts of SERA proteins remain covalently linked, while the central domain is released (Li *et al.*, 2002). The functions of the SERA N- and C-terminal domains remain unknown. It should be noted that the N- and C-terminal regions of *Pb*SERA1 and *Pb*SERA2 show only 34% and 29% amino acid sequence identity, respectively, whereas the central protease-like domain is more conserved (67% identity). Because in our mCherry constructs the fluorescent moiety is fused to the C-terminal end of SERA1 and SERA2, the fusion proteins only allow tracking of the C-terminal fragments of the corresponding SERA, and not the papain-like central domain after proteolytic processing.

Interestingly, by combining C-terminal tagging of *Pb*SERA2 and antibodies specific for the central domain of *Pb*SERA2, we found that the two domains localize to distinct compartments after formation of liver stage merozoites. While the C-terminal region of SERA2 remains associated with merozoites, the central putative papain-like domain localizes predominantly to the vacuolar compartment. This was also evidenced by differential Western blot analysis of vacuolar versus parasite protein fractions, which showed preferential accumulation of SERA2 processed forms (containing the central domain) in the vacuolar compartment of asexual blood stage parasites. This suggests that *Pb*SERA2 is proteolytically cleaved at the end of *Plasmodium* schizogony, the central domain being released in the vacuolar space while the C-terminus remains associated with merozoites.

Differently from *Pb*SERA1/mCherry, which was no longer detected after PVM rupture, *Pb*SERA2/mCherry was clearly found associated with liver and blood stage merozoites. This is reminiscent of *Pf*SERA5 localization on the surface of free merozoites after processing (Pang *et al.*, 1999; Okitsu *et al.*, 2007). Although the different expression patterns of *Pb*SERA1 and *Pb*SERA2 might be explained by differential stability of their N- and C-terminal domains after proteolytic processing, our observations raise the possibility that different SERAs fulfill different functions. *Pb*SERA1 and *Pb*SERA2 are particularly abundant in the final stages of liver and erythrocytic schizont maturation, which is compatible with a potential role in merozoite egress. Based on their differential localization, the two *P. berghei* SERAser may act at different steps of egress. In such a scenario, *Pb*SERA1, which localizes predominantly in the PV, could be involved in the rupture of the PVM. The *Pb*SERA2 C-terminal domain, which remains associated with merozoites after the PVM rupture, may rather play a role during subsequent steps, such as rupture of the host cell membrane or preparation of merozoites for invasion. Nevertheless, such roles, if any, can only be auxiliary, because parasites that lack *SERA1* and *SERA2* display no defects in egress of liver and blood stage merozoites, or natural malaria transmission in general.

So far, only *Pb*SERA5/ECP1 (the orthologue of *Pf*SERA8) was demonstrated to play a role during parasite egress. Interestingly, *Pb*SERA5/*Pf*SERA8 stands apart in the SERA family as it is expressed only in the mosquito stages and is the only SERA that apparently lacks a SUB1 cleavage site (Yeoh *et al.*, 2007). In this regard, it is noteworthy that inhibitors of *P. falciparum* SUB1, which cleaves (and presumably activates) SERAs, are more potent inhibitors of merozoite invasion than of merozoite egress (Yeoh *et al.*, 2007). While this observation could be due to different pharmacodynamic properties of the inhibitors, it is also compatible with a predominant role of SERAs during merozoite invasion of erythrocytes rather than parasite egress. The potential use of several invasion pathways may result in compensatory mechanisms explaining why single (and double) knockout parasites display no obvious defect during their progression through the life cycle. Because of the absence of detectable phenotype in parasites lacking *SERAser* genes, the loss-of-function approach cannot discriminate between a role of SERAser proteins during egress versus merozoite invasion. Gain-of-function approaches may constitute an alternative strategy, although it is probably difficult to overexpress *SERAser*, which are already highly expressed in normal parasites. Other strategies such as using blocking antibodies may help unraveling the function of the SERAser proteins.

Interestingly, we found that *P. berghei* parasites lacking both SERAser had increased levels of the cysteine-type SERA3, both at transcript and protein levels. Although we cannot formally exclude an effect of the modification *in cis* of the *SERA* gene locus in the knockout parasite lines, this observation suggests a potential functional link between SERAs belonging to distinct subfamilies. It has been shown before that *P. falciparum* parasites with a deletion of *PfSERA4* gene display increased RNA levels of *PfSERA5*, which is refractory to gene deletion (McCoubrie *et al.*, 2007). Similarly, attempts to knockout *SERA3* gene in *P. berghei* remained unsuccessful so far (E.D. Putrianti, F.F. Masduki, and K. Matuschewski, unpubl. data), suggesting that *PbSERA3* may play an essential role during *P. berghei* blood stage infection.

The crystal structure of the central protease-like domain of *Pf*SERA5 was recently solved, revealing several anomalies in the active site, in addition to the serine substitution (Hodder *et al.*, 2009). These structural features question the role of SERA5 as an actual protease, and may explain the limited proteolytic activity of recombinant *Pf*SERA5 *in vitro* (Hodder *et al.*, 2003). In the absence of a clear proteolytic activity of SERAser proteins under physiological conditions, multiple non-catalytic cellular roles, including regulatory functions, have to be considered. An attractive hypothesis is a potential function in substrate recognition. In analogy to ubiquitin E2-like variants of the ubiquitin/proteasome pathways that lack active site cysteine residues and form heterodimers with E2 enzymes (VanDemark *et al.*, 2001), SERAser, while catalytically inactive on their own, could act in concert with canonical SERAcys proteases and provide crucial substrate binding sites. In such a scenario, the diverse group of abundant and degenerate SERAser proteins would bind and recruit substrates to the proteases, thereby enhancing their overall proteolytic activity in the cell. While not essential, the presence of multiple substrate recognition proteins may greatly enhance the adjustment of the parasite to a changing environment prior to parasite exit of the respective host cell.

Importantly, a non-vital role *in vivo* does not exclude the potential of SERAser members for antimalaria subunit vaccine development. There is precedence for a nonessential target antigen, namely MSP3 (Mills *et al.*, 2002), which shows similar promising characteristics in a monkey challenge trial and functional assays with sera from immunized individuals (Hisaeda *et al.*, 2002; Druilhe *et al.*, 2005). The choice of candidate antigens for incorporation into a subunit vaccine does not necessitate an essential role in the parasite but should rather be based on functional assays (Matuschewski, 2006). Antibodies against *Pf*SERA5 inhibit parasite erythrocytic growth *in vitro* through agglutination of merozoites and ruptured schizonts (Pang *et al.*, 1999). Similarly to *Pf*SERA5, the C-terminal region of *Pb*SERA2 associates with merozoites, and therefore constitutes a potential target for inhibitory antibodies. In this regard, *P. berghei* mouse infection may thus represent a valuable *in vivo* model to evaluate antimalarial vaccines targeting SERA antigens. In contrast, target validation by reverse genetics to ultimately prove or disprove that loss of gene function results in non-viable malaria parasites *in vivo* is a prerequisite for preclinical development of tailor-made inhibitors against a target protein. Our results show that parasites grow normally in the absence of any *SERAser* members and cast profound doubt on the suitability to translate potential specific *Pf*SERA5 or other SERAser inhibitors into potent antimalarial drugs.

## Experimental procedures

### Experimental animals

Animals were from Charles River Laboratories. All animal work was conducted in accordance with European regulations and approved by the state authorities (Regierungspräsidium Karlsruhe).

### Reverse transcriptase PCR

Total RNA was purified from sporozoites, infected HuH7 cells or infected erythrocytes using the RNeasy kit (Qiagen). Reverse transcription was performed using the RETROscript kit (Ambion). cDNA was used as template for PCR amplification with primers specific for *P. berghei SERA1* (forward, GTAACTGGACAAGAAGAAACACAAG; reverse, CAGGATTGATAGCATAAACTCTTGAAC), *SERA2* (forward, GGTACTCCAGATATGATAGTAAATATAATTGG; reverse, GGCCCTGATCCGGAAGATGAACCTGAAGG), *TRAP* (forward, CCCGGATCCATGAAGCTCTTAGGAAATAG; reverse, GTGTGGATCCTTCCTGACAAACTTTAGAAAG), *GAPDH* (forward, ATGGCAATAACAAAAGTCGGAATTAATGG; reverse, TGTGGATAGCCAAATCTAAAAGACGG), *MSP1* (forward, CTGGTTTGGTAGGAGAAGGCGAATC; reverse, AGCTACAGAATACACCATCATAAT). Real-time qPCR was performed on cDNA preparations from mixed blood stages, purified blood stage schizonts or late liver stages in HuH7 cell cultures, using the ABI 7500 sequence detection system and Power SYBR Green PCR Master Mix (Applied Biosystems), according to the manufacturer's instructions. qPCR was performed in triplicates, with 1 cycle of 95°C for 15 min, followed by 40 cycles of 95°C for 15 s, 55°C for 15 s and 60°C for 45 s. Standard curves were generated for all primers using WT cDNA serial dilutions and gave amplification efficiencies of 90–100%. Data were analysed with the SDS 1.3.1 software (Applied Biosystems). Relative transcript abundance was normalized to *MSP1* expression. The following primers were used for real-time PCR: *SERA1* (forward, CAAGTGGGTATAAGACTAGAATTTATGC; reverse, AATTGGCATATACTCCATTTCGCAGC), *SERA2* (forward, ACACAAGGTCAAGCCCCACAAAGCC; reverse, CCCACCATTTTCTGGAGTTTCAACG), *SERA3* (forward, GTTGATGTTTTAGGTCCAGATAATTGTG; reverse, GTGGTAAAAATTTGAACTGAGTTGTGG), *SERA4* (forward, CAATTCAGAAAAAAATAGACATTACCC; reverse, TTTAAACCAATACTTTGTACCCTCGAAC), *SERA5* (forward, CCAAACTGATTTGACTGTAACTATAGG; reverse, ATAACTTCACTGTGCATGCATGTGTCG), *MSP1* (forward, AAATAAATCTGGTTTGGTAGGAGAAGG; reverse, CCGCAGTTTGACAACCAGCAGTTGG).

### Generation of the mCherry-tagged *SERA1* and *SERA2* parasite lines

For targeted fluorescent tagging of *SERA1* and *SERA2*, an integration vector was generated by amplification of a PCR fragment using *P. berghei* genomic DNA as template and primers mCherry-SERA1for (5′-ATAAGAATGCGGCCGCTACCACATGAGAATGAATTTGCAGGG-3′; NotI site is underlined) and mCherry-SERA1rev (5′-GGACTAGTCACATAACAAAAGTAGCAATCGTCTG-3′; SpeI site is underlined), or mCherry-SERA2for (5′-ATAAGAATGCGGCCGCATGTTGGTGATTCATGCCCCG-3′; NotI site is underlined) and mCherry-SERA2rev (5′-TGCTCTAGATACAGCGCAAAAGTTACATTCATTATCACC-3′; XbaI site is underlined). Cloning into the *P. berghei* transfection vector that contained the mCherry sequence and *PbDHFR/TS* 3′ UTR resulted in plasmids pEDP05 and pEDP06, for tagging of *Pb*SERA1 and *Pb*SERA2 respectively. The targeting plasmids were linearized with HpaI and AarI respectively, and parasite transfection, positive selection, and parasite cloning was performed as described previously (Janse *et al.*, 2006). Integration-specific PCR amplification of the mCherry-tagged *SERA1* or *SERA2* was generated using specific primer combinations. We obtained one parasite population each that was used for a systematic expression and localization analysis. Expression of the mCherry fusion proteins was analysed through direct detection of the red fluorescence of mCherry by confocal microscopy. Hoechst 33342 (Molecular Probes) was used to stain nuclei. Images were acquired on a Zeiss LSM510 confocal system (Zeiss, Germany) equipped with visible and UV laser lines, and processed with Adobe Photoshop software (Adobe Systems).

### Generation of anti-*Pb*SERA1 and anti-SERA2 antibodies

DNA fragments corresponding to the coding sequence of *Pb*SERA1 C-terminal region (Leu^877^-Ser^1122^) and *Pb*SERA2 central (M) domain (Lys^546^-Pro^714^) were amplified from *P. berghei* cDNA by RT-PCR and cloned into pGEX6P-1 vector (Amersham, Buckinghampshire, England). Recombinant proteins were expressed in *Escherichia coli* BL21 cells (Stratagene) as glutathione S-transferase (GST) fusion proteins, and purified using glutathione-agarose as described by the manufacturer (Amersham Biosciences). Purified proteins were used to immunize Lewis rats along with complete Freund adjuvant, followed by multiple boosting immunizations. A rat monoclonal antibody was generated against *Pb*SERA1-C. To this purpose, B cells were isolated from lymph nodes of one rat immunized against *Pb*SERA1-C protein and fused to the mouse myeloma cell line P3X63.Ag8.653. The positive pools of hybridoma cells reacting with SERA1-C were screened by indirect ELISA. Single-cell clones were isolated by limited dilution, leading to the isolation of clone C65 (SERA1C), which was characterized further and found to bind an epitope (D892-EPASISTQ-E901) at the C-terminus of *Pb*SERA1.

### Western blot analysis

Parasite protein extracts were obtained from *P. berghei* Nycodenz-enriched blood stage schizont preparations, after lysis in saponin followed by Triton X-100, to differentiate the vacuolar compartment from the parasite fraction, as described (Schmidt-Christensen *et al.*, 2008). Proteins were separated on 10% SDS-PAGE reducing gels and transferred to PVDF membranes (Amersham). Membranes were probed with anti-SERA1 and anti-SERA2 rat antibodies, anti-SERA3 mouse antibodies (Schmidt-Christensen *et al.*, 2008) or anti-HSP70 mouse antibodies (Tsuji *et al.*, 1994). Horseradish peroxidase-conjugated goat anti-rat or anti-mouse antibodies (Sigma) were used for detection, and bands were visualized by enhanced chemiluminescence (Amersham).

### Immunofluorescence assay

For analysis of *Pb*SERAser localization in late liver stages, infected HepG2 cells were fixed with 4% paraformaldehyde, permeabilized with ice-cold methanol and incubated with primary antibodies against *Pb*SERA1C or *Pb*SERA2M (rat). A chicken anti-EXP1 antibody was used to stain the PVM. Bound antibodies were detected using anti-rat Alexa Fluor 594- or anti-chicken Cy5- conjugated secondary antibodies (Molecular Probes, Leiden, the Netherlands). Nuclei were visualized with DAPI (Sigma-Aldrich, Germany). Immunofluorescence labelled cells were examined by confocal microscopy using the Olympus FV1000 (SIM scanner and spectral detection).

### Generation of the *sera*_ser_ knockout parasite lines

For targeted replacement of *PbSERA1*, a replacement vector was generated by amplification of two PCR fragments using *P. berghei* genomic DNA as template and primers SERA1_forI (5′-GGGGTACCCCCATACCATCACCCCCTTCAAC-3′; KpnI site is underlined) and SERA1_revII (5′-GCCCAAGCTTCCAGTTCTCCCGTACCTTCAACCACC-3′; HindIII site is underlined) to amplify the 5′ flanking region, and SERA1_forIII (5′-CGCGGATCCGTTGGCAAAGGGGAATATATCGTATCA-3′; BamHI site is underlined) and SERA1_revIV (5′-TCCCCGCGGCGAATTTTTACAACTTAAACCATAGTGCAC-3′; SacII site is underlined) for the 3′ flanking region respectively. Similarly, for replacement of *PbSERA2* we employed primers SERA2_forI (5′-GGGGTACCGAACCGTTTTTAGGTCGATACGTTCTGTGCTG-3′; KpnI site is underlined) and SERA2_revII (5′-GCCCAAGCTTCCCATATATTGTTTGACGAACAAAATAC-3′; HindIII site is underlined) as well as SERA2_forIII (5′-GGACTAGTGGTTCATCTTCCGGATCAGGGCCAACACCTT-3′; SpeI site is underlined) and SERA2_revIV (5′-TCCCCGCGGTTGAGATTGGGGGCATGCTTTTATTACCA-3′, SacII site is underlined). The targeting vector to generate the *SERA1/2* double mutant was cloned from the 5′ flanking region of SERA1 and 3′ flanking region of SERA2 fragments. Cloning into the *P. berghei* transfection vector (Thathy and Ménard, 2002) resulted in plasmids pEDP01, pEDP02 and pEDP03 for p*SERA1*(−), p*SERA2*(−) and p*SERA1*(−)*/2*(−) respectively. The targeting plasmids were linearized with KpnI/SacII, and parasite transfection, positive selection and parasite cloning were performed as described previously (Janse *et al.*, 2006). Transfections were performed in the *P. berghei* ANKA strain, except for *sera2*(−) parasites, which were generated in the *P. berghei* NK65 strain. Replacement-specific PCR amplifications of the corresponding *SERA_ser_*(−) loci were generated using specific primer combinations. We obtained four, five and four independent *sera1*(−), *sera2*(−), and *sera1*(−)*/2*(−) clonal parasite populations, respectively, that were phenotypically identical. Detailed analysis was performed with one representative clone each.

### Phenotypical analysis during the *Plasmodium* life cycle *in vivo*

Blood stage development was analysed *in vivo* in asynchronous infections using NMRI mice. Gametocyte differentiation and exflagellation of microgametes were detected in mice before mosquito feedings. *Anopheles stephensi* mosquito rearing and maintenance was carried out under a 14 h light/10 h dark cycle, 75% humidity and at 28°C or 20°C respectively. Sporozoite populations were separated and analysed as described previously (Vanderberg, 1975). For determination of sporozoite infectivity, and numbers of midgut- and salivary gland-associated sporozoites, infected mosquitoes were dissected at days 10, 14 and 17 after feeding respectively. For determination of the infectivity of sporozoites, infected mosquitoes were dissected at day 17 after feeding. Sporozoites were liberated from salivary glands and injected intravenously at the numbers indicated into young Sprague/Dawley (SD) rats. Patency was checked daily by Giemsa-stained blood smears.

### *In vitro* experiments

For analysis of gliding motility, sporozoites isolated from infected mosquito salivary glands were deposited on glass slides coated with bovine serum albumin, and incubated at 37°C for 30 min. Trails left behind gliding parasites were then visualized using anti-CSP antibodies (Potocnjak *et al.*, 1980). For analysis of EEF development, we used HuH7 cells cultured in DMEM supplemented with 10% FCS and antibiotics. *P. berghei* sporozoites were added in triplicate wells, incubated for 2 h at 37°C, and washed off. After 24 or 48 h, EEFs were revealed using primary antibodies against *Plasmodium* heat shock protein 70 (HSP70) (Tsuji *et al.*, 1994).

## Supporting information

**Fig. S1.** Genotyping of *PbSERA1/mCherry* (A) and *PbSERA2/mCherry* (B) clonal parasites. Confirmation of the predicted integration events by diagnostic PCR fragments, tests 1–4 (see Fig. 3A). Absence of the WT signal confirmed purity of the clonal parasite lines.

**Fig. S2.** Processed forms of *Pb*SERA2 localize preferentially to the PV compartment. Purified schizonts from WT *P. berghei* parasites were lysed in saponin (PV fraction), followed by Triton X-100 (parasite fraction). Protein extracts were analysed by Western blot using rat antibodies specific for the central domain of *Pb*SERA2. Processed forms of SERA2 enriched in the PV fraction are indicated with an arrowhead, whereas other specific bands are shown with an arrow.

**Fig. S3.** Immunofluorescence analysis confirms the absence of *SERA1* and *SERA2* expression in *P. berghei sera1*(−)*/2*(−) parasites. HepG2 cells infected with *P. berghei* WT or *sera1*(−)*/2*(−) parasites at the cytomere stage were stained with antibodies against PbSERA1 (A) or PbSERA2 (B). PVM was stained with anti-EXP1 antibodies (cyan), and DNA was labelled with DAPI (blue). Scale bar: 5 μm.
