# Supplementary figures and images for: The Plasmodium serine-type SERA proteases display distinct expression patterns and non-essential in vivo roles during life cycle progression of the malaria parasite
Source: Cell Microbiol. 2010 Jan 20;12(6):725–39. doi: 10.1111/j.1462-5822.2009.01419.x (PMC2878606; doi:10.1111/j.1462-5822.2009.01419.x)

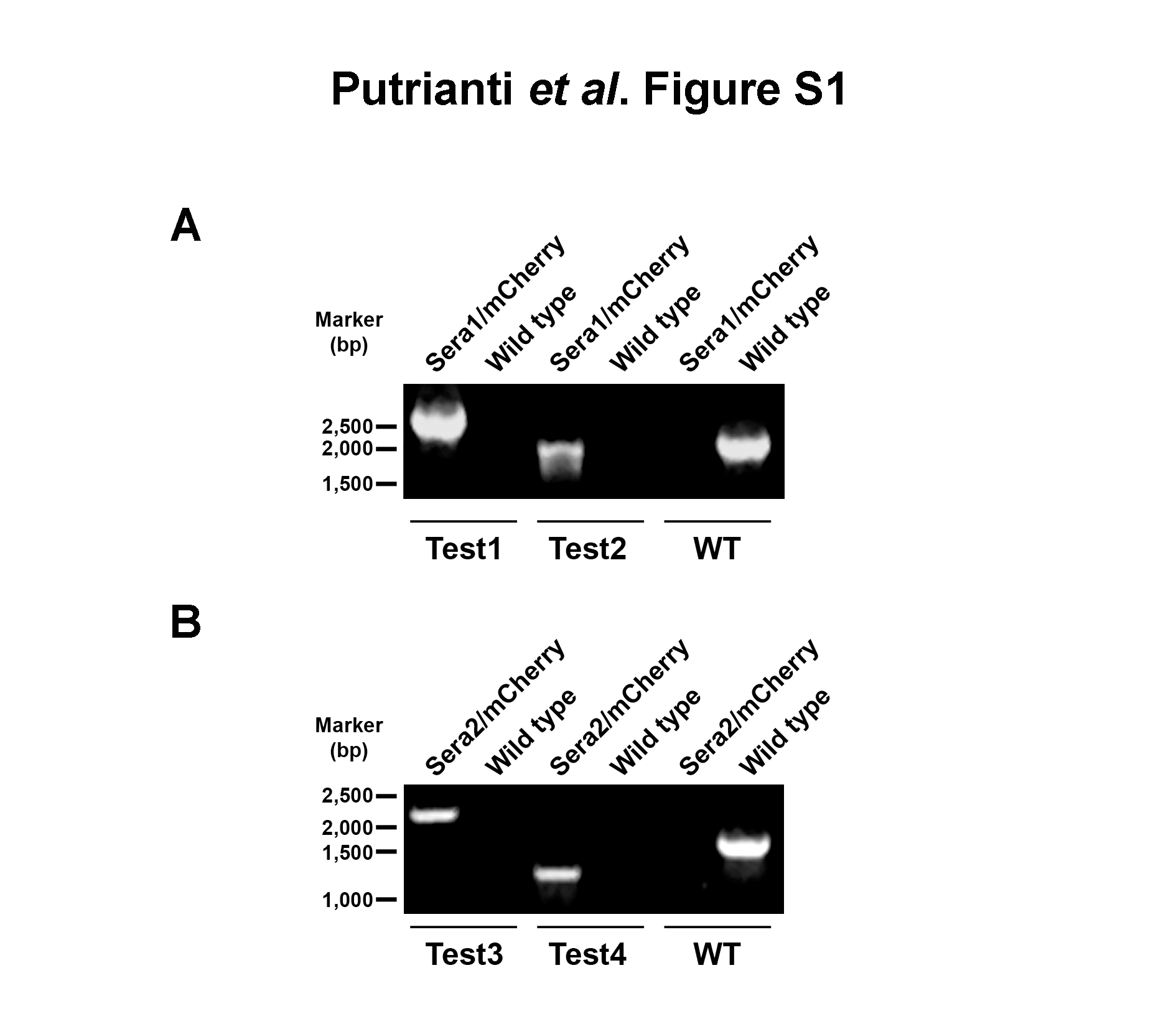

Supplement: Supplementary file 1 [file cmi0012-0725-SD1.tif]

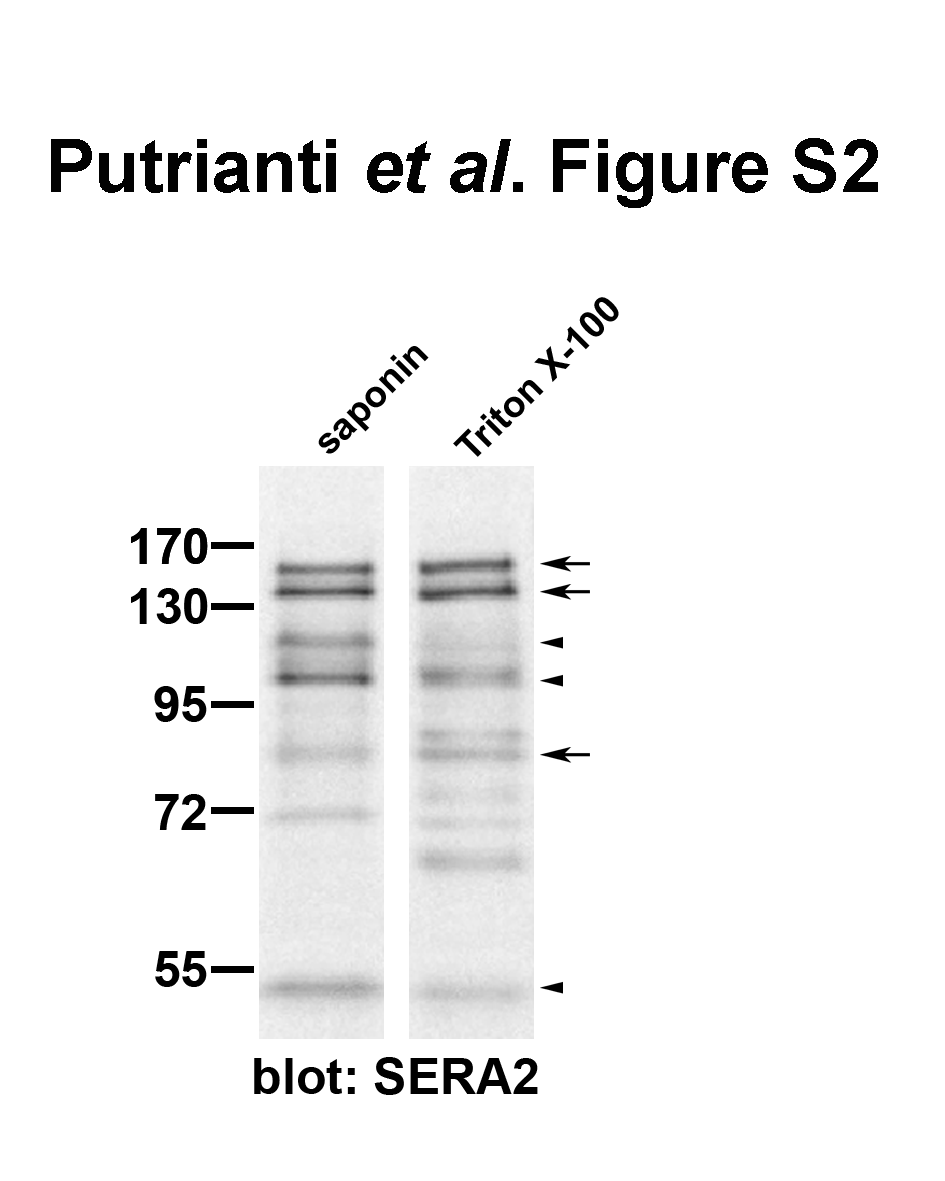

Supplement: Supplementary file 2 [file cmi0012-0725-SD2.tif]

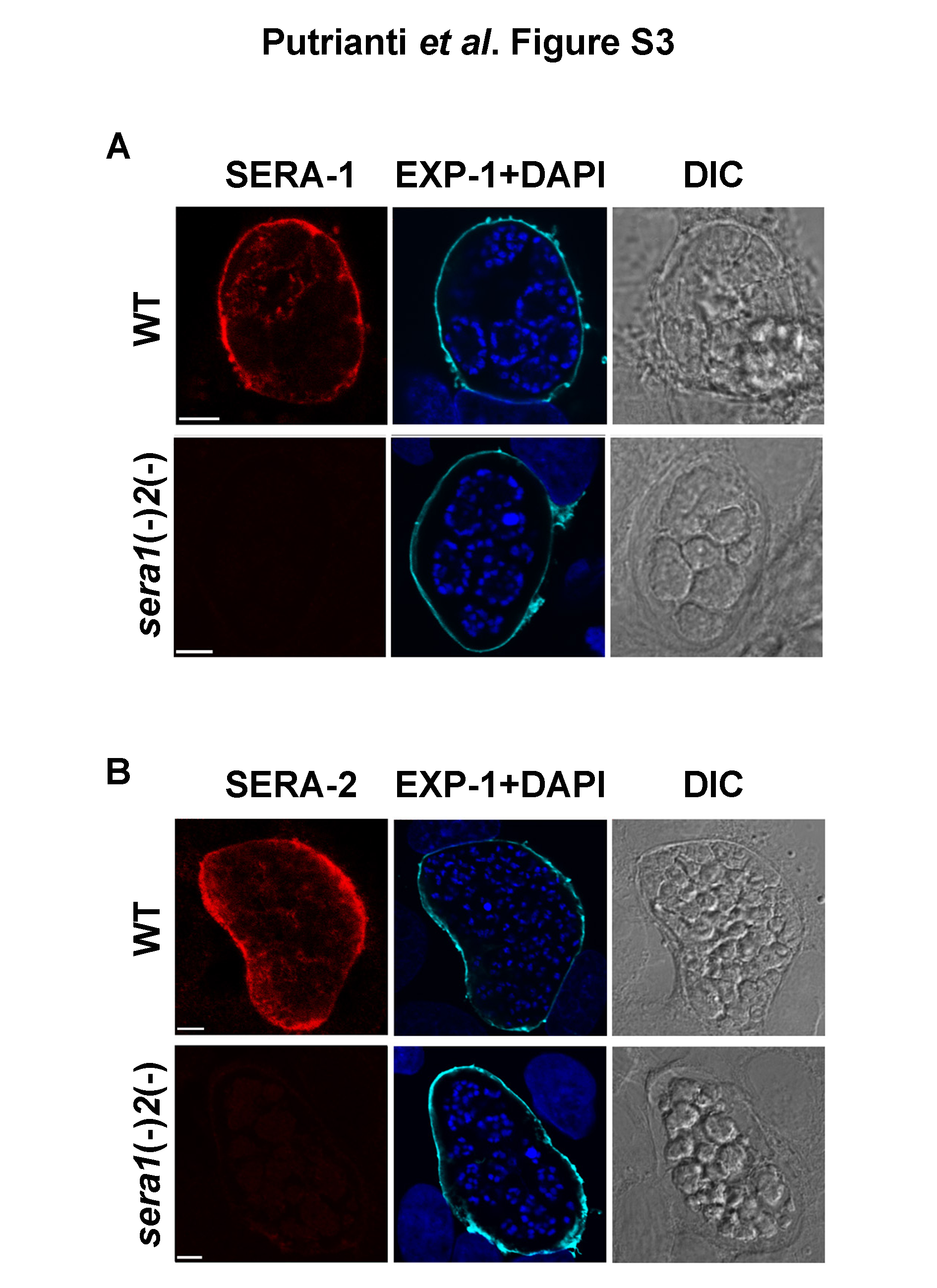

Supplement: Supplementary file 3 [file cmi0012-0725-SD3.tif]
